# iBitter-Fuse: A Novel Sequence-Based Bitter Peptide Predictor by Fusing Multi-View Features

**DOI:** 10.3390/ijms22168958

**Published:** 2021-08-19

**Authors:** Phasit Charoenkwan, Chanin Nantasenamat, Md. Mehedi Hasan, Mohammad Ali Moni, Pietro Lio’, Watshara Shoombuatong

**Affiliations:** 1 Modern Management and Information Technology, College of Arts, Media and Technology, Chiang Mai University, Chiang Mai 50200, Thailand; phasit.c@cmu.ac.th; 2 Center of Data Mining and Biomedical Informatics, Faculty of Medical Technology, Mahidol University, Bangkok 10700, Thailand; chanin.nan@mahidol.edu; 3 Tulane Center for Biomedical Informatics and Genomics, John W. Deming Department of Medicine, Division of Biomedical Informatics and Genomics, School of Medicine, Tulane University, New Orleans, LA 70112, USA; mhasan1@tulane.edu; 4 Faculty of Health and Behavioural Sciences, School of Health and Rehabilitation Sciences, The University of Queensland, St Lucia, QLD 4072, Australia; m.moni@uq.edu.au; 5 Department of Computer Science and Technology, University of Cambridge, Cambridge CB3 0FD, UK; pl219@cam.ac.uk

**Keywords:** bitter peptide, bioinformatics, support vector machine, feature selection, machine learning, classification

## Abstract

Accurate identification of bitter peptides is of great importance for better understanding their biochemical and biophysical properties. To date, machine learning-based methods have become effective approaches for providing a good avenue for identifying potential bitter peptides from large-scale protein datasets. Although few machine learning-based predictors have been developed for identifying the bitterness of peptides, their prediction performances could be improved. In this study, we developed a new predictor (named iBitter-Fuse) for achieving more accurate identification of bitter peptides. In the proposed iBitter-Fuse, we have integrated a variety of feature encoding schemes for providing sufficient information from different aspects, namely consisting of compositional information and physicochemical properties. To enhance the predictive performance, the customized genetic algorithm utilizing self-assessment-report (GA-SAR) was employed for identifying informative features followed by inputting optimal ones into a support vector machine (SVM)-based classifier for developing the final model (iBitter-Fuse). Benchmarking experiments based on both 10-fold cross-validation and independent tests indicated that the iBitter-Fuse was able to achieve more accurate performance as compared to state-of-the-art methods. To facilitate the high-throughput identification of bitter peptides, the iBitter-Fuse web server was established and made freely available online. It is anticipated that the iBitter-Fuse will be a useful tool for aiding the discovery and de novo design of bitter peptides.

## 1. Introduction

To protect themselves from environmental toxins, mammalian species, including humans, are averse to bitter-tasting substances. [1]. A bitter peptide is well-known for its ability to interact with bitter taste receptors (T2Rs) in the oral cavity [2,3]. The ability of a peptide to be bitter is determined by its amino acid composition; hydrophobic amino acids are especially known for their bitter characteristics. It is therefore important to investigate and characterize the bitterness intensity as they play an important role for drug development and nutritional research [4,5,6,7]. Although experimental methods are considered to be reliable approaches for characterizing the bitterness of peptides [5,8,9], they are usually time-consuming and expensive. Due to their convenience and high efficiency, machine-learning (ML) methods have attracted increasing attention in the field of bioinformatics. Thus far, several computational methods based on quantitative structure–activity relationship (QSAR) modeling have been published on the prediction of peptide bitterness [10,11,12,13,14,15]. For instance, Yin et al. [12] generated a collection of QSAR models in order to estimate the bitterness of dipeptides. Specifically, support vector regression (SVR) was used for constructing QSAR models for analyzing 48 angiotensin-converting enzyme (ACE) inhibitor dipeptides, 55 ACE inhibitor tripeptides and 48 bitter dipeptides. In addition, the quantitative multidimensional amino acid descriptors E (E1–E5) were introduced in this aspect, where E1, E2, E3, E4 and E5 represents hydrophobicity, steric properties or side chain bulk/molecular size, preferences for amino acids to occur in α-helices, composition and the net charge, respectively. In 2016, Huang et al. [6] introduced BitterX, which is the first online available tool developed for identifying human bitter taste receptors. In BitterX, sequential minimal optimization (SMO), logistic regression (LR) and random forest (RF) were employed to develop ML-based models in order to discriminate bitter from non-bitter compounds. In their experimental setting, training (70%) and hold-out test (30%) datasets were constructed for model development and validation. Performance as evaluated in terms of accuracy (ACC) was 0.93 and 0.83 for training and hold-out test sets, respectively. Subsequently, BitterPredict was developed by Dagan-Wiener et al. [16] in order to identify bitter compounds based on the information of chemical structures.

In this study, we focused on the identification of bitter peptides based on sequence information. According to our research, only two ML-based prediction tools have been published to identify bitter peptides (iBitter-SCM [17] and BERT4Bitter [18]). The first sequence-based bitter peptide predictor was introduced by Charoenkwan et al. and is called iBitter-SCM [17]. iBitter-SCM used propensity scores of peptides for predicting and analyzing the bitterness of peptides. In addition, these propensity scores were employed to provide better understanding on biochemical and biophysical properties of bitter peptides. Recently, deep learning (DL) algorithms were considered to develop the prediction model in this aspect. The same group presented BERT4Bitter, which is based on the bidirectional encoder representation from transformers (BERT)-based predictor for the prediction of bitter peptides. Although iBitter-SCM [17] and BERT4Bitter [18] could yield reasonably high prediction accuracies, there remain certain shortcomings. Firstly, the generalization capability of ML-based predictors will depend on the feature representation method. However, iBitter-SCM [17] employed only dipeptide composition (DPC) for representing peptide sequences, which was unable to fully capture the discriminative characteristics between bitter and non-bitter peptides [19,20,21,22,23,24]. Secondly, the embodiment of non-important features in model development might have led to two possible outcomes: information redundancy and over-fitting [23,24,25,26,27,28,29,30]. Finally, the overall performance of existing methods is not yet of satisfactory level.

**Figure 1:**
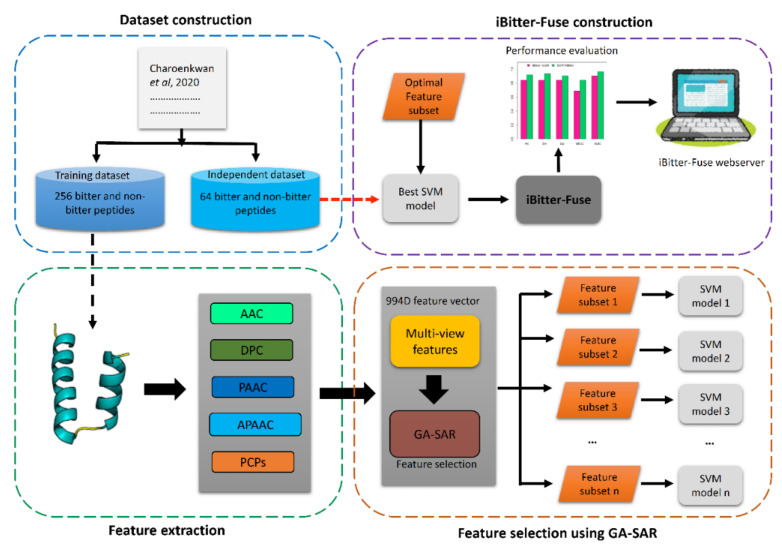
Schematic framework of iBitter-Fuse for predicting bitter peptides. The main procedure in the design of iBitter-Fuse is essentially comprised of the following steps: (**i**) dataset construction, (**ii**) feature extraction, (**iii**) feature selection using GA-SAR and (**iv**) iBitter-Fuse construction.

Motivated by these considerations, we present iBitter-Fuse, which is a novel computational model designed for accurate and large-scale identification of bitter peptides. The schematic framework of iBitter-Fuse for bitter peptide identification is depicted in Figure 1. Particularly, we explored a variety of feature encoding schemes (e.g., DPC, amino acid composition (AAC), pseudo amino acid composition (PAAC), amphiphilic pseudo amino acid composition (APAAC) and physicochemical properties from AAindex (AAI)) for providing sufficient information from different aspects (i.e., pertaining to compositional information and physicochemical properties) to build a more comprehensive prediction model. To enhance the predictive performance, the customized genetic algorithm utilizing self-assessment-report (GA-SAR) as introduced by Charoenkwan et al. [26] was employed in identifying *m* informative features and the optimal ones are used as input to the support vector machine (SVM)-based classifier for development of the final model (iBitter-Fuse). Extensive comparative analysis indicated that the proposed iBitter-Fuse, which only utilizes *m* = 36 selected features, was able to achieve significantly better performance than those of conventional ML classifiers as evaluated by 10-fold cross-validation and independent tests. Moreover, iBitter-Fuse was shown to outperform existing state-of-the-art predictors in terms of ACC (0.930), Sn (0.938), Sp (0.922) and MCC (0.859) as evaluated on the independent test. Results highlighted that the proposed iBitter-Fuse has better generalization capability and discriminative power for accurately identifying bitter peptides than that of existing methods and conventional ML classifiers. Finally, the predictive model was deployed as the iBitter-Fuse web server and made freely available online at http://camt.pythonanywhere.com/iBitter-Fuse (accessed on 8 August 2021).

## 2. Materials and Methods

### 2.1. Benchmark Dataset

The same benchmark dataset called BTP640 [17,18] was used to develop and evaluate our proposed predictor. This dataset had 640 peptide sequences that consisted of 320 bitter peptides and 320 non-bitter peptides. To create a fair test, the BTP640 dataset was randomly divided into training and independent test datasets using a ratio of 8:2. Finally, the training dataset consisted of 256 bitter peptides and 256 non-bitter peptides, while the independent test dataset consisted of 64 bitter peptides and 64 non-bitter peptides. Further details about this benchmark dataset can be found in [17]. It should be noted that both of these datasets can be retrieved from http://pmlab.pythonanywhere.com/BERT4Bitter (accessed on 1 July 2021).

### 2.2. Feature Encodings

AAC and DPC represent the proportions of each amino acid and dipeptide in a peptide sequence **P** that are expressed as fixed lengths of 20 and 400, respectively. Thus, in terms of AAC and DPC features, a peptide **P** can be expressed by vectors with 20D and 400D (dimension) spaces, respectively, as formulated by:

(1) $$P={\left[{\mathrm{aa}}_{1},{\mathrm{aa}}_{2},\ldots ,\;{\mathrm{aa}}_{20}\right]}^{T}$$

(2) $$P={\left[{\mathrm{dp}}_{1},{\mathrm{dp}}_{2},\ldots ,\;{\mathrm{dp}}_{400}\right]}^{T}$$

where T is the transposed operator, while aa_1_, aa_2_…, aa_20_ and dp_1_, dp_2_…, dp_400_ are occurrence frequencies of the 20 and 400 native amino acids and dipeptides, respectively, in a peptide sequence **P**.

There are 544 AAIs of amino acids derived from version 9.0 of the Amino acid index database (AAindex) [31]. Each AAI consisted of a set of 20 numerical values for amino acids where AAIs having NA values were discarded. Finally, 531 AAIs (531D) were used in the extraction of features from peptide sequences that are then used for the development of models in this study. Previously, AAIs have been regarded as one of the most intuitive features associated with biophysical and biochemical reactions and is also referred to as an easy and interpretable feature [17,32,33,34,35,36,37,38,39].

As mentioned in previous studies [40,41,42] and shown in Equations (3) and (4), AAC and DPC features only provide compositional information of a peptide sequence, but all of the sequence-order information may be completely lost. To remedy this limitation, PAAC and APAAC approaches were proposed by Chou [41]. According to Chou’s PAAC, the general form of PAAC for a peptide **P** is formulated by:

(3) $$P={\left[\Psi_{1},\Psi_{2},\ldots ,\Psi_{u},\ldots ,\;\Psi_{\Omega }\right]}^{T}$$

where the subscript Ω is an integer to reflect the feature’s dimension. The value of Ω and the component of $\Psi_{u}$ where u=1,2,…,Ω is dependent on protein or peptide sequences. In this study, parameters of PAAC (i.e., the discrete correlation factor λ and weight of the sequence information ϖ) were estimated by using the optimization procedure as described hereafter. The dimension of PAAC feature is 20 + λ ×ϖ. Since the hydrophobic and hydrophilic properties of proteins plays an important role in their folding and interaction, APAAC was introduced by Chou [41]. The dimension of APAAC feature is 20 + 2λ. Particularly, the first 20 components are the 20 basic AAC ($p_{1},p_{2},\ldots ,{\;p}_{20}$), while the next 2λ ones denote the set of correlation factors that reveal physicochemical properties such as hydrophobicity and hydrophilicity in a protein or peptide sequence as formulated by:

(4) $$Ρ={\left[p_{1},p_{2},\ldots ,{\;p}_{20},p_{20+\lambda },p_{20+\lambda +1},\ldots p_{20+2\lambda }\right]}^{T}$$

Parameters of PAAC (ω^1^ and λ^1^) and APAAC (ω^2^ and λ^2^) were optimized by varying weight and lambda values from 0 to 1 and 1 to 10 with step sizes of 0.1 and 1, respectively, on the training dataset as evaluated by the 10-fold CV test. Subsequently, all parameter sets of PAAC and APAAC were used to individually develop SVM-based classifier and the parameter set provided the best cross-validation ACC, which was considered as the optimal set. More details on how to estimate such parameters can be found elsewhere [19,20]. In addition, PAAC and APAAC descriptors are described in the Supplementary Materials. After performing such parameter optimization, ω^1^, λ^1^, ω^2^ and λ^2^ were found to be 0.7, 1, 0.2 and 1, respectively.

### 2.3. Support Vector Machine

SVM is an effective ML algorithm for dealing with binary classification problem and has been widely used in various biological problems [30,43,44,45,46,47,48]. This method is based on the Vapnik–Chervonenkis theory of statistical learning [49,50,51]. Briefly, SVM constructs a binary classifier by determining a separate hyper plane with the largest distance between two classes. In order to make linear separation on high dimensional samples, SVM employs a well-known kernel function for transforming the sample space having *p*-dimensional feature vector onto a feature space with *n*-dimensional feature vector where *p < n*. In this work, the widely used radial basis function is applied to non-linearly transform the feature space as defined as follows:

(5) $$K\left(x_{i},x_{j}\right)=\exp\left(-\gamma ∥x_{i}-x_{j}∥{}^{2}\right),\;\gamma >0$$

The kernel parameter γ represents how samples are transformed to the feature space while the cost parameter C of SVM model adjusts the penalty of the total error. To enhance the performance of the SVM model, *C* and γ parameters were tuned by using the grid search method and evaluated via the 10-fold cross-validation scheme using the training dataset where the search space for *C* and γ are [2^−2^, 2^2^] and [2^−2^, 2^2^] with steps of 2 and 2, respectively.

### 2.4. Feature Selection Based on GA-SAR

To save time and computational resources, a wise approach is to use a feature selection algorithm for identifying informative features [23,24,26,44,52,53,54,55,56,57,58]. In this study, the GA-SAR algorithm was utilized to determine the minimal number of informative features while maximizing performance results of the SVM model [26]. The original GA-SAR was first proposed by Charoenkwan et al. [26] for the improved prediction of quorum-sensing peptides. In GA-SAR, a self-assessment-report (SAR) approach is utilized to construct a profile used for reporting the usefulness of a feature pool based on the assumption that a good feature will be highly correlated with the output variable, but poorly correlated to each other. More specifically, the GA-SAR algorithm can automatically rank the most informative features and simultaneously estimate SVM’s parameters. Therefore, the chromosome of GA-SAR comprises of the parameter setting of two main genes: (i) binary genes for the purpose of informative features selection and (ii) parametric genes for the purpose of the SVM’s parameter optimization. Herein, 994 binary genes contain two 3-bit for encoding C (2^−2^, 2^−1^, …, 2^2^) and γ (2^−2^, 2^−1^, …, 2^2^) parameters of the SVM model. More details on the GA-SAR algorithm are described in our previous studies [19,20,26].

### 2.5. Performance Evaluation

In order to evaluate the prediction ability of the model, we used four widely used metrics for the two-class prediction problem as follows:

(6) $$\mathrm{ACC}=\frac{\mathrm{TP}+\mathrm{TN}}{\left(\mathrm{TP}+\mathrm{TN}+\mathrm{FP}+\mathrm{FN}\right)}$$

(7) $$\mathrm{Sn}=\frac{\mathrm{TP}}{\left(\mathrm{TP}+\mathrm{FN}\right)}$$

(8) $$\mathrm{Sp}=\frac{\mathrm{TN}}{\left(\mathrm{TN}+\mathrm{FP}\right)}$$

(9) $$\mathrm{MCC}=\frac{\mathrm{TP}\times \mathrm{TN}-\mathrm{FP}\times \mathrm{FN}}{\sqrt{\left(\mathrm{TP}+\mathrm{FP}\right)\left(\mathrm{TP}+\mathrm{FN}\right)\left(\mathrm{TN}+\mathrm{FP}\right)\left(\mathrm{TN}+\mathrm{FN}\right)}}$$

where ACC, Sn, Sp and MCC represent the accuracy, sensitivity, specificity and Matthews correlation coefficient, respectively. TP and TN indicate the number of correctly predicted true bitter peptides and true non-bitter peptides, respectively. Meanwhile, FP indicates the number of non-bitter peptides predicted as bitter peptides and FN indicates the number of bitter peptides predicted as non-bitter peptides. Model comparison of the proposed model with those of previously described models was performed via the use of the receiver operating characteristic (ROC) curve of threshold-independent parameters. Correspondingly the area under the ROC curve (AUC) was utilized to assess the prediction performance whereby AUC values in the range of 0.5 and 1 are indicative of random and perfect models, respectively.

## 3. Results and Discussion

### 3.1. Performance Comparison of Different Feature Encodings

**Figure 2:**
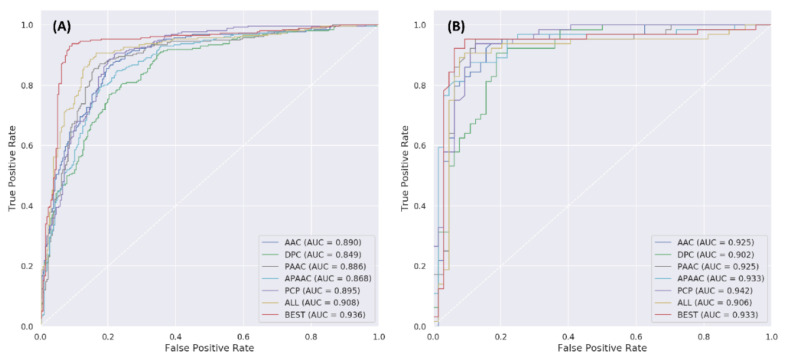
ROC curves of different feature encodings based on 10-fold cross-validation (**A**) and independent tests (**B**).

In this study, five well-known feature encodings (i.e., AAC, DPC, PAAC, APAAC and AAI) and the fused feature (i.e., AAC + DPC + PAAC + APAAC + AAI) were used for training predictive models using the SVM method for accurately predicting bitter peptides. Table 1 and Figure 2 show their 10-fold cross-validation and independent test results. Based on cross-validation results, it was observed that the highest ACC, MCC and AUC of 86.72%, 0.736 and 0.903, respectively, was achieved by using the fused feature, while the second and third highest performance results were achieved by using PAAC and AAI descriptors. Interestingly, Figure 2A shows that the fused feature also exhibits a great capability to identify bitter peptides with an AUC of 0.903 and achieves the best performance on the training dataset among other types of feature encodings. As for the independent dataset, Table 1 and Figure 2B show that the overall prediction performance is quite consistent with the 10-fold cross-validation results. The fused feature still provided the highest performance in terms of three out of five performance metrics (i.e., ACC, Sn and MCC). Specifically, the fused feature provided ACC of 0.906, Sn of 0.922 and MCC of 0.813 as well as an AUC of 0.906.

### 3.2. Determination of Optimal Features

As mentioned above, the fused feature outperformed the five feature encodings. Based on the fused feature scheme, each peptide sequence was represented with a 994D feature vector, but the number of sequences used in the training dataset was 512. This problem might cause two outcomes: information redundancy and over-fitting. To address this issue, the GA-SAR was employed to identify *m* out of 994 features followed by simultaneous tuning of SVM parameters where the number of *m* is in the range of 10–50; the search range of SVM parameter is recorded in Supplementary Table S1. We hypothesized that if a feature is selected by GA-SAR, it is considered to be beneficial for the bitter peptide prediction [19,20,26]. Due to the non-deterministic characteristics of GA-SAR, ten individual experiments were performed to generate ten different feature sets. Specifically, these ten feature sets were individually used as the input feature to individually construct ten SVM models whose corresponding 10-fold cross-validation and independent test results are recorded in Table 2 and Table 3.

From Table 2, it can be noticed that the three top-ranking feature sets (ACC) are comprised of feature sets from experiments 1 (0.920), 2 (0.918) and 3 (0.912), respectively. The feature set from experiment 2 achieved very comparable results to those from experiment 1. In the case of the independent test results as recorded in Table 3, it can be observed that the three top-ranked feature sets were from experiment 2 (0.923), 10 (0.914) and 9 (0.914), respectively. Taking into consideration 10-fold cross-validation and independent test results, the feature set from experiment 2 was considered as the optimal one and was used for developing the proposed iBitter-Fuse; more details of this feature set are summarized in Table 4 and Supplementary Table S3. Furthermore, the selected feature set consisted of m = 36 informative features covering four different feature descriptors: AAC (4 features), DPC (13 features), PAAC (1 feature) and AAI (18 features).

### 3.3. Comparison of Our Fused Features and Other Feature Descriptors

**Figure 3:**
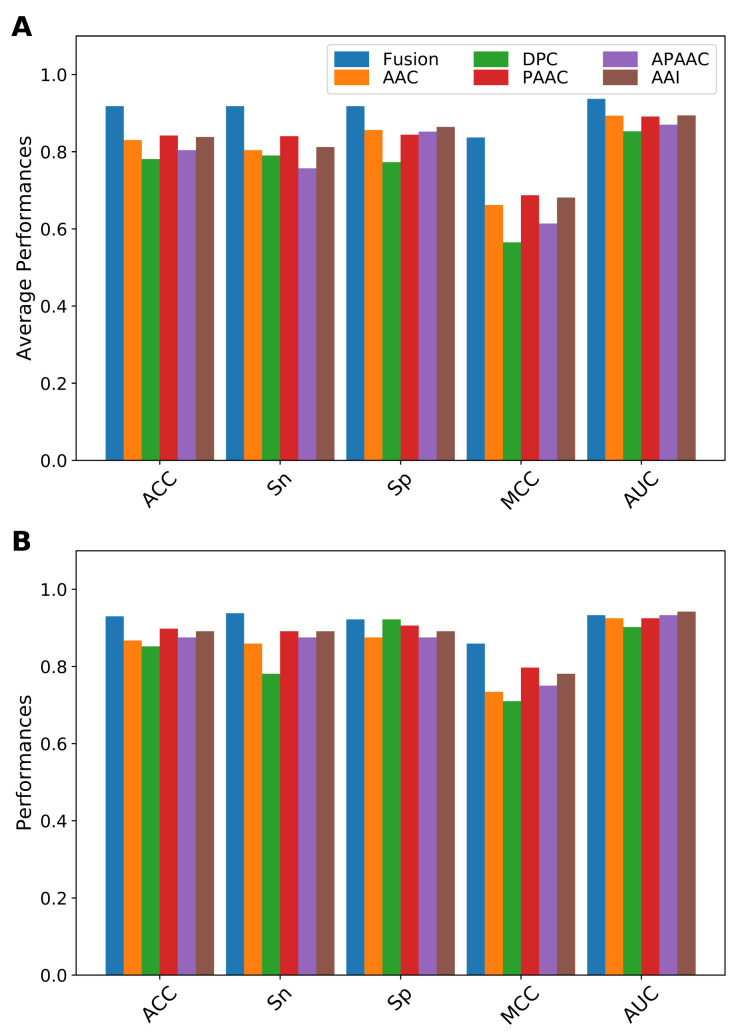
Performance evaluations of our fused features and the five individual feature descriptors based on (**A**) 10-fold cross-validation and (**B**) independent tests.

In this section, we compared the performance of our fused features (the selected *m* = 36 informative features) with the five individual feature descriptors (AAC, DPC, PAAC, APAAC and AAI). The performance of our fused features and the compared feature descriptors is summarized in Figure 3 and Supplementary Table S2. As shown in Figure 3A, it can be observed that the 10-fold cross-validation results of our fused features were significantly better than that of the five selected feature descriptors when evaluated from all five performance metrics. Specifically, the ACC, Sn and MCC of our fused features were 0.918, 0.918 and 0.837, respectively, where 7.6–13.7%, 7.8–16.1% and 15–22.7% were higher than other feature descriptors. Regarding independent test results, the performance of our fused features was still better than those of other feature descriptors as evaluated by ACC (0.930), Sn (0.938), Sp (0.922) and MCC (0.859) (Figure 3B). This demonstrated that the fusion of different view information was effective in contributing to the improved predictive performance.

### 3.4. Comparison of iBitter-Fuse with Conventional ML Classifiers

**Figure 4:**
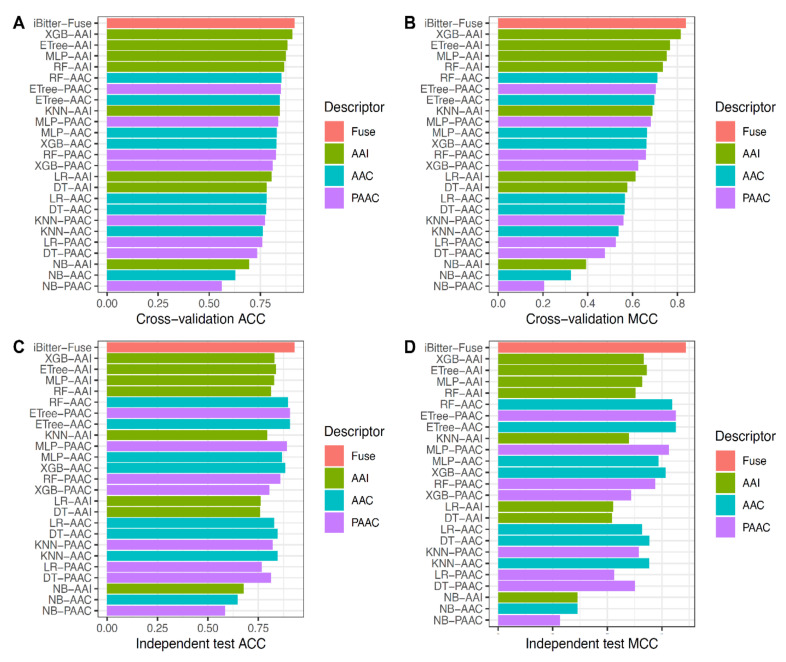
Performance evaluations of iBitter-Fuse and different ML classifiers based on (**A**,**B**) their 10-fold cross-validation ACC and MCC and (**C**,**D**) independent test ACC and MCC.

**Figure 5:**
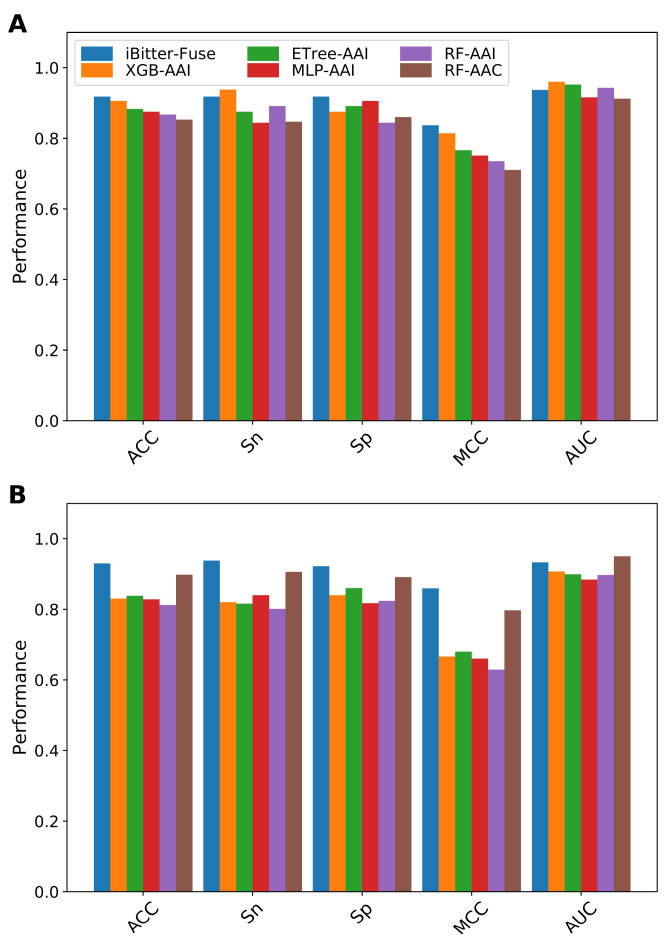
Performance evaluations of iBitter-Fuse and top five ML classifiers based on (**A**) 10-fold cross-validation and (**B**) independent tests.

To evaluate the predictive performance of iBitter-Fuse, we compared its performance with those of conventional ML classifiers. Herein, we constructed and optimized several ML classifiers trained using decision tree (DT), extremely randomized trees (ETree), k-nearest neighbor (KNN), multi-layer perceptron (MLP), naive Bayes (NB), random forest (RF) and extreme gradient boosting (XGB) with three selected feature descriptors (AAC, AAI and PAAC). All of these ML classifiers were constructed and optimized using the *scikit-learn* Python machine-learning package (version 0.22) [59]. Herein, the optimal hyperparameters of respective ML classifiers were determined by using a grid search procedure and 10-fold cross-validation scheme (i.e., the search range is presented in Supplementary Table S1). To make a fair test, the same training and independent test datasets were used for model training and validation, respectively. Figure 4 and Supplementary Tables S4 and S5 display the details of 10-fold cross-validation and independent test results of iBitter-Fuse and several ML classifiers. Furthermore, Figure 5 and Table 5 show the performance comparison of our predictor with those of top five ML classifiers (XGB-AAI, ETree-AAI, MLP-AAI, RF-AAI and RF-AAC).

As can be seen from Figure 5, we observe that the 10-fold cross-validation results of our predictor is better than the five selected feature descriptors in terms of three out of five performance metrics (ACC, Sp and MCC). To be specific, our predictor obtained the maximum ACC of 0.918, Sp of 0.918 and MCC of 0.837, which were 1.2%, 4.3% and 2.3% higher than the best ML classifier (XGB-AAI) (Table 5). Remarkably, our predictor showed 2–10.6% higher ACC, 7.8–11.7% higher Sn, 2.7–10.1% higher Sp and 4–20.8% higher MCC than top five ML classifiers on the independent test dataset, indicating that the proposed iBitter-Fuse was more capable for identifying bitter peptides than those of conventional ML classifiers as developed in this study (Figure 4 and Figure 5).

### 3.5. Comparison of iBitter-Fuse with the State-of-the-Art Methods

To evaluate the effectiveness of the iBitter-Fuse, we compared its performance with state-of-the-art methods, namely iBitter-SCM [17] and BERT4Bitter [18]. Of note, the proposed iBitter-Fuse and state-of-the-art methods were developed and evaluated using the same benchmark dataset. The performance of iBitter-SCM and BERT4Bitter was obtained directly from the published work of BERT4Bitter [18]. Table 6 records the 10-fold cross-validation and independent test results of iBitter-Fuse and state-of-the-art methods. In the case of 10-fold cross-validation results, iBitter-Fuse clearly outperformed existing methods in terms of four out of five performance metrics (ACC, Sp, MCC and AUC) based on the 10-fold cross-validation test. Particularly, ACC, Sp, MCC and AUC of iBitter-Fuse were approximately 74.7–5.7%, 6.4–9%, 8.6–11.1% and 2.2–3.4% higher than that of existing methods. To further validate the robustness of iBitter-Fuse, the approach was tested and compared with existing methods by using the independent test dataset. As can be seen from Table 6, our predictor significantly outperforms iBitter-SCM in achieving 8.6%, 9.4%, 7.8% and 17.1% improvement in terms of ACC, Sn, Sp and MCC, respectively. Meanwhile, our predictor could achieve slightly better performance than that of BERT4Bitter as evaluated by four out of five performance metrics (ACC, Sp, Sn and MCC). Taking into consideration the cross-validation and independent test results, the aforementioned comparative results indicated that the proposed iBitter-Fuse was more precise and stable for the identification of bitterness of peptides than those of existing predictors.

### 3.6. iBitter-Fuse Web Server

**Figure 6:**
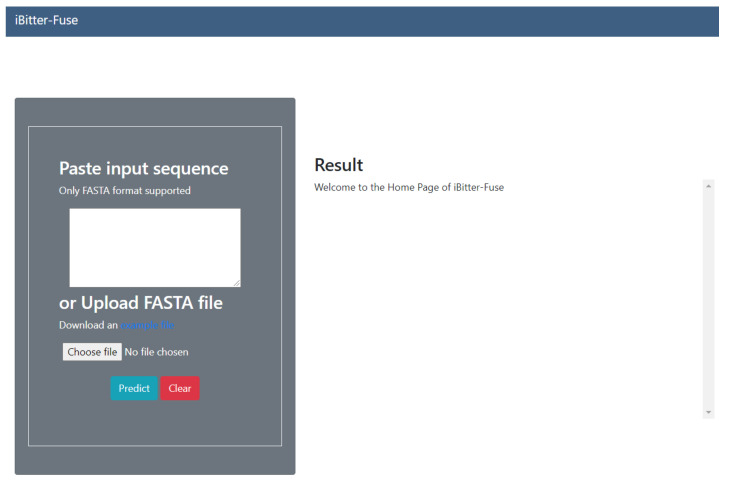
Screenshots of the iBitter-Fuse web server.

In order to allow easy access by the scientific community, we have developed a user-friendly web server iBitter-Fuse that is freely available online at http://camt.pythonanywhere.com/iBitter-Fuse (accessed on 8 August 2021). Herein, we provide step-by-step guidelines on how to use the iBitter-Fuse web server. Firstly, users can access the web server by entering the URL mentioned in the previous sentence, which would bring us to the webpage as shown in Figure 6. Secondly, the user can enter the query sequence into the text box or upload a FASTA file by clicking on the “Choose file” button. Thirdly, the user can click on the “Submit” button in order to start the prediction process; this step typically takes a few seconds for the server to process the task. Finally, after finishing the prediction process, results are displayed on the right-hand side of the web server. Examples of FASTA-formatted sequences can be accessed by clicking on the “example file” button.

## 4. Conclusions

A novel computational model referred herein as iBitter-Fuse was developed for accurately identifying the bitterness of peptides. In the development of iBitter-Fuse, we have explored a variety of feature encoding schemes for providing sufficient information from different aspects, including compositional information and physicochemical properties. Subsequently, the optimal feature set was determined by using the GA-SAR approach and used as input to the SVM-based classifier for development of a more robust model. We have conducted extensive benchmarking experiments via both 10-fold cross-validation and independent tests. Extensive comparative analysis indicated that the proposed iBitter-Fuse was more effective and could outperform conventional ML classifiers as well as existing state-of-the-art predictors as evaluated by 10-fold cross-validation and independent tests. This thereby highlights the effectiveness and generalization capability of the proposed iBitter-Fuse. Finally, for the convenience of experimental scientists, the iBitter-Fuse web server was established and made freely available online at http://camt.pythonanywhere.com/iBitter-Fuse (accessed on 8 August 2021). We believe that the iBitter-Fuse may serve as a useful and cost-effective approach for predicting bitter peptides on a large scale as well as facilitating de novo bitter peptide design.

## Figures and Tables

**Table 1:** Cross-validation and independent test results of different feature encodings.

Cross-Validation	Feature	#Feature	ACC	Sn	Sp	MCC	AUC
10-fold CV	AAC	20	0.830	0.804	0.856	0.662	0.893
	DPC	400	0.781	0.790	0.773	0.565	0.853
	PAAC	21	0.842	0.840	0.844	0.687	0.891
	APAAC	22	0.804	0.757	0.852	0.614	0.870
	AAI	531	0.838	0.812	0.864	0.681	0.894
	Fusion	994	0.867	0.855	0.879	0.736	0.911
Independent test	AAC	20	0.867	0.859	0.875	0.734	0.925
	DPC	400	0.852	0.781	0.922	0.710	0.902
	PAAC	21	0.898	0.891	0.906	0.797	0.925
	APAAC	22	0.875	0.875	0.875	0.750	0.933
	AAI	531	0.891	0.891	0.891	0.781	0.942
	Fusion	994	0.906	0.922	0.891	0.813	0.906

**Table 2:** Cross-validation results of ten SVM models trained with various feature sets derived from GA-SAR.

#Exp.	#Feature ^a^	ACC	Sn	Sp	MCC	AUC
1	37	0.920	0.898	0.941	0.842	0.947
2	36	0.918	0.918	0.918	0.837	0.937
3	36	0.912	0.910	0.914	0.825	0.945
4	41	0.910	0.906	0.914	0.822	0.924
5	36	0.906	0.914	0.899	0.814	0.937
6	40	0.906	0.902	0.910	0.814	0.950
7	38	0.906	0.890	0.922	0.814	0.925
8	37	0.898	0.898	0.899	0.802	0.932
9	36	0.896	0.871	0.922	0.795	0.947
10	38	0.896	0.906	0.887	0.795	0.938
Mean		0.907	0.901	0.913	0.816	0.938
STD.		0.008	0.013	0.015	0.016	0.009

^a^ #Feature represents the number of features used for constructing a model. Experiment #2 afforded the optimal prediction performance and is therefore used for further analysis.

**Table 3:** Independent test results of ten SVM models trained with various feature sets derived from GA-SAR.

#Exp.	#Feature ^a^	ACC	Sn	Sp	MCC	AUC
1	37	0.891	0.875	0.906	0.782	0.935
2	36	0.930	0.938	0.922	0.859	0.933
3	36	0.891	0.906	0.875	0.782	0.925
4	41	0.898	0.906	0.891	0.797	0.922
5	36	0.883	0.906	0.859	0.766	0.930
6	40	0.898	0.891	0.906	0.797	0.926
7	38	0.906	0.938	0.875	0.814	0.949
8	37	0.891	0.859	0.922	0.783	0.938
9	36	0.914	0.922	0.906	0.828	0.939
10	38	0.914	0.953	0.875	0.831	0.935
Mean		0.902	0.909	0.894	0.804	0.933
STD.		0.014	0.029	0.022	0.029	0.008

^a^ #Feature represents the number of features used for constructing a model. Experiment #2 having the optimal prediction performance is used for further analysis.

**Table 4:** List of fused informative features having *m* = 36 features as derived from the GA-SAR algorithm.

Feature	#Feature	List
AAC	4	I, K, W, Y
DPC	13	AA, AF, EL, GV, IA, IQ, KG, LE, LQ, PF, QL, TD, YG
PAAC	1	Xc1.P
AAI	18	BIGC670101, DESM900101, FAUJ880106, FAUJ880110, GOLD730101, GRAR740102, NAKH900113, OOBM770104, QIAN880129, VENT840101, WERD780102, WOLS870103, YUTK870102, ZIMJ680103, MUNV940105, TAKK010101, CEDJ970102, HARY940101

**Table 5:** Performance comparison of iBitter-Fuse with top five ML classifiers.

Cross-Validation	#Feature	ACC	Sn	Sp	MCC	AUC
10-fold CV	iBitter-Fuse	0.918	0.918	0.918	0.837	0.937
	XGB-AAI	0.906	0.938	0.875	0.814	0.960
	ETree-AAI	0.883	0.875	0.891	0.766	0.952
	MLP-AAI	0.875	0.844	0.906	0.751	0.916
	RF-AAI	0.867	0.891	0.844	0.735	0.943
	RF-AAC	0.853	0.847	0.86	0.71	0.912
Independent test	iBitter-Fuse	0.930	0.938	0.922	0.859	0.933
	XGB-AAI	0.830	0.820	0.840	0.666	0.907
	ETree-AAI	0.838	0.816	0.860	0.680	0.899
	MLP-AAI	0.828	0.840	0.817	0.660	0.884
	RF-AAI	0.812	0.801	0.824	0.629	0.897
	RF-AAC	0.898	0.906	0.891	0.797	0.950

**Table 6:** Performance comparison of iBitter-Fuse with the existing methods.

Cross-Validation	Classifier ^a^	ACC	Sn	Sp	MCC	AUC
10-fold CV	iBitter-SCM	0.871	0.913	0.828	0.751	0.903
	BERT4Bitter	0.861	0.868	0.854	0.726	0.915
	iBitter-Fuse	0.918	0.918	0.918	0.837	0.937
Independent test	iBitter-SCM	0.844	0.844	0.844	0.688	0.904
	BERT4Bitter	0.922	0.938	0.906	0.844	0.964
	iBitter-Fuse	0.930	0.938	0.922	0.859	0.933

^a^ Results come from the work BERT4Bitter [16].

## Data Availability

All the data are available at http://pmlab.pythonanywhere.com/dataset.

## Supplementary Materials

The following are available online at https://www.mdpi.com/article/10.3390/ijms22168958/s1.
