# Supplementary material for: iBitter-Fuse: A Novel Sequence-Based Bitter Peptide Predictor by Fusing Multi-View Features
Source: Int J Mol Sci. 2021 Aug 19;22(16):8958. doi: 10.3390/ijms22168958 (PMC8396555; doi:10.3390/ijms22168958)
Supplement: Supplementary file 1 [file ijms-22-08958-s001.zip › ijms-1312297-supplementary.pdf]

PAAC descriptor (or the type 1 PAAC) consists of 20 + λ discrete features [1], where the first 20 features are the classical AAC descriptor and sequence order-correlated factors are defined as follows:

$$\begin{cases} \theta_1 = \dfrac{1}{N-1}\displaystyle\sum_{i=1}^{N-1}\Theta(R_i, R_{i+1}) \\ \theta_2 = \dfrac{1}{N-2}\displaystyle\sum_{i=1}^{N-2}\Theta(R_i, R_{i+2}) \\ \theta_3 = \dfrac{1}{N-3}\displaystyle\sum_{i=1}^{N-3}\Theta(R_i, R_{i+3}) \\ \cdots \\ \cdots \\ \theta_\lambda = \dfrac{1}{N-1}\displaystyle\sum_{i=1}^{N-\lambda}\Theta(R_i, R_{i+\lambda}) \end{cases} \quad (1)$$

where λ ($\lambda < L$) is a parameter to be specified and L is the protein/peptide length. Meanwhile, θ_1 is the 1st rank coupling factor encoding the sequence-order correlation between all the most contiguous residues along a protein chain, θ_2 is the 2nd rank coupling factor encoding the sequence-order correlation between all the 2nd most contiguous residues, θ_3 is the 3rd rank coupling factor encoding the sequence-order correlation between all the 3rd most contiguous residues and so forth. And $\Theta(\mathrm{R}_i, \mathrm{R}_j)$, which is a set of N amino acid properties, it can be defined as: where $H_k(\mathrm{R}_i)$ is the kth property in the amino acid property set for amino acid R_i,can be represented by

$$\Theta(\mathrm{R}_i, \mathrm{R}_j) = \frac{1}{N}\sum_{K=1}^{N}[H_k(\mathrm{R}_i) - H_k(\mathrm{R}_j)]^2 \quad (2)$$

where $H_k(\mathrm{R}_i)$ is the kth property in the amino acid property set for amino acid R_i,defined as follows:

$$H_k(\mathrm{R}_i) = \frac{H_k^o - \frac{1}{20}\sum_{i=1}^{20} H_k^o(\mathrm{R}_i)}{\sqrt{\frac{\sum_{i=1}^{20}[H_k^o(\mathrm{R}_i) - \frac{1}{20}\sum_{i=1}^{20} H_k^o(\mathrm{R}_i)]^2}{20}}} \quad (3)$$

Where H_1^o, H_2^o and H_3^o represent hydrophobicity value, hydrophilicity value and side chain mass, respectively, of the amino acid. Let f_i is the normalized frequency of natural 20 amino acids in the protein sequence. Thus, a set of the 20 + λ discrete features or PAAC can be defines as:

$$P = \begin{bmatrix} x_1 \\ x_2 \\ \cdots \\ x_{20} \\ x_{20+1} \\ \cdots \\ x_{20+\lambda} \end{bmatrix} \quad (4)$$

$$x_c = \frac{f_c}{\sum_r^{20} f_r + \omega\sum_j^{\lambda}\theta_j} \qquad (1 < c < 20) \quad (5)$$

$$x_c = \frac{\omega\theta_{c-20}}{\sum_r^{20} f_r + \omega\sum_j^{\lambda}\theta_j} \qquad (21 < c < 20 + \lambda) \quad (6)$$

where θ_j is the jth rank sequence-coupling factor based on Eqs. (1) and (3) and f_i (i = 1,2,…,20) is the normalized frequency of natural 20 amino acids in the protein sequence. The λ and ω are important of PAAC descriptor.

APAAC descriptor is known as the type 2 PAAC [1] and its definition is similar to the PAAC descriptor. As mentioned above, H_1^o and H_2^o represent hydrophobicity value and hydrophilicity value, and the hydrophobicity and hydrophilicity correlation functions are defined as:

$$H_{i,j}^1 = H_1(i)H_1(j) \tag{7}$$

$$H_{i,j}^2 = H_2(i)H_2(j) \tag{8}$$

The sequence order-correlated factors are defined as follows:

$$\begin{cases} \tau_1 = \frac{1}{N-1}\sum_{i=1}^{N-1} H_{I,I+1}^1 \\ \tau_2 = \frac{1}{N-1}\sum_{i=1}^{N-1} H_{I,I+1}^2 \\ \tau_3 = \frac{1}{N-2}\sum_{i=1}^{N-2} H_{I,I+2}^1 \\ \tau_4 = \frac{1}{N-2}\sum_{i=1}^{N-2} H_{I,I+2}^2 \\ \quad\cdots \\ \quad\cdots \\ \tau_{2\lambda-1} = \frac{1}{N-\lambda}\sum_{i=1}^{N-\lambda} H_{I,I+\lambda}^1 \\ \tau_{2\lambda} = \frac{1}{N-\lambda}\sum_{i=1}^{N-\lambda} H_{I,I+\lambda}^2 \end{cases} \tag{9}$$

APAAC descriptor is represented with a set of the 20 + 2λ discrete features, defined as follows:

$$P = \begin{bmatrix} x_1 \\ x_2 \\ \cdots \\ x_{20} \\ x_{20+1} \\ \cdots \\ x_{20+\lambda} \\ x_{20+\lambda+1} \\ x_{20+\lambda+2} \\ \cdots \\ x_{20+2\lambda} \end{bmatrix} \tag{10}$$

$$x_c = \frac{f_c}{\sum_r^{20} f_r + \omega \sum_j^{2\lambda} \tau_j} \qquad (1 < c < 20) \tag{11}$$

$$x_c = \frac{w\tau_{c-20}}{\sum_r^{20} f_r + \omega \sum_j^{2\lambda} \tau_j} \qquad (21 < c < 20 + 2\lambda) \tag{12}$$

where f_i (i = 1,2,…,20) is the normalized frequency of natural 20 amino acids in the protein sequence, τ_j the j-tier sequence-correlation factor computed according to Eq. (9) and ω is important of APAAC descriptor.

Table S1. Hyperparameter search details for six populart ML algorithms.

Method	Parameters	Range of parameters
ETree	n_estimators	[20, 50, 100, 200, 500]
LR	C	[0.001, 0.01, 0.1, 1, 10, 100, 1000]
MLP	hidden_layer_sizes	[1.0, 2.0, 4.0, 8.0, 16.0, 32.0, 64.0, 128.0, 256.0]
RF	n_estimators	[20, 50, 100, 200, 500]
	max_features	sqrt(n_features)
SVM	penalty parameter (C[a])	[2^{-2}–2^2] in $\log_2$ steps
	kernel coefficient (γ[a])	[2^{-2}–2^2] in $\log_2$ steps
XGB	n_estimators	[20, 50,100,200,500]

Columns 2 and 3 represent the parameter name used in the Scikit-learn library and the range of parameter used to develop the model, respectively.

Table S2. Performance comparison of our proposed multi-view features with five well-known feature descriptors.

Cross-validation	Feature	#Feature	ACC	Sn	Sp	MCC	AUC
10-fold CV	Fusion	36	0.918	0.918	0.918	0.837	0.937
	AAC	20	0.830	0.804	0.856	0.662	0.893
	DPC	400	0.781	0.790	0.773	0.565	0.853
	PAAC	21	0.842	0.840	0.844	0.687	0.891
	APAAC	22	0.804	0.757	0.852	0.614	0.870
	AAI	531	0.838	0.812	0.864	0.681	0.894
Independent test	Fusion	36	0.930	0.938	0.922	0.859	0.933
	AAC	20	0.867	0.859	0.875	0.734	0.925
	DPC	400	0.852	0.781	0.922	0.710	0.902
	PAAC	21	0.898	0.891	0.906	0.797	0.925
	APAAC	22	0.875	0.875	0.875	0.750	0.933
	AAI	531	0.891	0.891	0.891	0.781	0.942

Table S3 Summary information of the selected 36 informative features derived from GA-SAR.

Feature	Type of Descriptor	Description
I	AAC	Frequency of I presenting in a protein sequence
K	AAC	Frequency of K presenting in a protein sequence
W	AAC	Frequency of W presenting in a protein sequence
Y	AAC	Frequency of Y presenting in a protein sequence
AA	DPC	Frequency of AA presenting in a protein sequence
AF	DPC	Frequency of AF presenting in a protein sequence
EL	DPC	Frequency of EL presenting in a protein sequence
GV	DPC	Frequency of GV presenting in a protein sequence
IA	DPC	Frequency of IA presenting in a protein sequence
IQ	DPC	Frequency of IQ presenting in a protein sequence
KG	DPC	Frequency of KG presenting in a protein sequence
LE	DPC	Frequency of LE presenting in a protein sequence
LQ	DPC	Frequency of LQ presenting in a protein sequence
PF	DPC	Frequency of PF presenting in a protein sequence
QL	DPC	Frequency of QL presenting in a protein sequence
TD	DPC	Frequency of TP presenting in a protein sequence
YG	DPC	Frequency of YG presenting in a protein sequence
Xc1.P	PAAC	Frequency of P presenting in a protein sequence
BIGC670101	AAI	Residue volume (Bigelow, 1967)
DESM900101	AAI	Hydrophobicity index, 3.0 pH (Cowan-Whittaker, 1990)
FAUJ880106	AAI	STERIMOL maximum width of the side chain (Fauchere et al., 1988)
FAUJ880110	AAI	Number of full nonbonding orbitals (Fauchere et al., 1988)
GOLD730101	AAI	Hydrophobicity factor (Goldsack-Chalifoux, 1973)
GRAR740102	AAI	Polarity (Grantham, 1974)
NAKH900113	AAI	Ratio of average and computed composition (Nakashima et al., 1
OOBM770104	AAI	Average non-bonded energy per residue (Oobatake-Ooi, 1977)
QIAN880129	AAI	Weights for coil at the window position of -4 (Qian-Sejnowski, 19
VENT840101	AAI	Bitterness (Venanzi, 1984)
WERD780102	AAI	Free energy change of epsilon(i) to epsilon(ex) (Wertz-Scheraga, 1978)
WOLS870103	AAI	Principal property value z3 (Wold et al., 1987)
YUTK870102	AAI	Unfolding Gibbs energy in water, pH9.0 (Yutani et al., 1987)
ZIMJ680103	AAI	Polarity (Zimmerman et al., 1968)
MUNV940105	AAI	Free energy in beta-strand region (Munoz-Serrano, 1994)
TAKK010101	AAI	Side-chain contribution to protein stability (kJ/mol) (Takano-Yutani, 2001)
CEDJ970102	AAI	Composition of amino acids in anchored proteins (percent) (Cedano et al., 1997)
HARY940101	AAI	Mean volumes of residues buried in protein interiors (Harpaz et al., 1994)

Table S4 Cross-validation results of ML-based classifiers built eight ML methods with three feature descriptors.

Descriptor	Classifier	Parameter	ACC	Sn	Sp	MCC	AUC
AAC	DT	N/A	0.779	0.816	0.742	0.563	0.781
	ETree	50	0.846	0.847	0.844	0.697	0.903
	KNN	N/A	0.762	0.836	0.688	0.537	0.762
	LR	100	0.781	0.789	0.774	0.565	0.824
	MLP	300	0.830	0.847	0.813	0.664	0.882
	NB	N/A	0.627	0.933	0.320	0.325	0.817
	RF	500	0.853	0.847	0.860	0.710	0.912
	XGB	100	0.828	0.836	0.821	0.661	0.898
PAAC	DT	N/A	0.734	0.796	0.672	0.476	0.742
	ETree	300	0.850	0.851	0.848	0.704	0.909
	KNN	N/A	0.773	0.824	0.723	0.558	0.773
	LR	10	0.760	0.738	0.782	0.525	0.811
	MLP	50	0.838	0.859	0.816	0.682	0.895
	NB	N/A	0.562	0.953	0.172	0.205	0.798
	RF	100	0.826	0.859	0.793	0.659	0.900
	XGB	300	0.809	0.855	0.762	0.625	0.885
AAI	DT	N/A	0.781	0.891	0.672	0.576	0.788
	ETree	500	0.883	0.875	0.891	0.766	0.952
	KNN	N/A	0.844	0.859	0.828	0.688	0.844
	LR	1	0.805	0.859	0.750	0.613	0.855
	MLP	100	0.875	0.844	0.906	0.751	0.916
	NB	N/A	0.695	0.656	0.734	0.392	0.789
	RF	100	0.867	0.891	0.844	0.735	0.943
	XGB	100	0.906	0.938	0.875	0.814	0.960

Table S5. Independent test results of ML-based classifiers built eight ML methods with three feature descriptors.

Descriptor	Classifier	Parameter	ACC	Sn	Sp	MCC	AUC
AAC	DT	N/A	0.844	0.906	0.781	0.693	0.842
	ETree	50	0.906	0.906	0.906	0.813	0.958
	KNN	N/A	0.844	0.891	0.797	0.691	0.844
	LR	100	0.828	0.875	0.781	0.659	0.867
	MLP	300	0.867	0.891	0.844	0.735	0.919
	NB	N/A	0.648	0.938	0.359	0.364	0.848
	RF	500	0.898	0.906	0.891	0.797	0.950
	XGB	100	0.883	0.891	0.875	0.766	0.932
PAAC	DT	N/A	0.813	0.844	0.781	0.626	0.819
	ETree	300	0.906	0.891	0.922	0.813	0.957
	KNN	N/A	0.820	0.859	0.781	0.643	0.820
	LR	10	0.766	0.766	0.766	0.531	0.863
	MLP	50	0.891	0.906	0.875	0.782	0.947
	NB	N/A	0.586	0.984	0.188	0.284	0.868
	RF	100	0.859	0.844	0.875	0.719	0.935
	XGB	300	0.805	0.797	0.813	0.609	0.907
AAI	DT	N/A	0.758	0.797	0.718	0.522	0.762
	ETree	500	0.838	0.816	0.860	0.680	0.899
	KNN	N/A	0.793	0.840	0.747	0.599	0.794
	LR	1	0.762	0.770	0.754	0.527	0.827
	MLP	100	0.828	0.840	0.817	0.660	0.884
	NB	N/A	0.678	0.585	0.770	0.363	0.699
	RF	100	0.812	0.801	0.824	0.629	0.897
	XGB	100	0.830	0.820	0.840	0.666	0.907

[1] K. C. Chou, "Prediction of protein cellular attributes using pseudo-amino acid composition," *Proteins: Structure, Function, and Bioinformatics*, vol. 43, no. 3, pp. 246-255, 2001.
